# Extracellular Vesicles from Ocular Melanoma Have Pro-Fibrotic and Pro-Angiogenic Properties on the Tumor Microenvironment

**DOI:** 10.3390/cells11233828

**Published:** 2022-11-29

**Authors:** Léo Piquet, Kelly Coutant, Andrew Mitchell, Amel Ben Anes, Enola Bollmann, Nathan Schoonjans, Julie Bérubé, François Bordeleau, Alain Brisson, Solange Landreville

**Affiliations:** 1 Faculté de Médecine, Université Laval, Quebec City, QC G1V 0A6, Canada; 2 Centre de Recherche du CHU de Québec-Université Laval, Quebec City, QC G1S 4L8, Canada; 3 Centre de Recherche sur le Cancer de l’Université Laval, Quebec City, QC G1R 3S3, Canada; 4 Centre de Recherche en Organogénèse Expérimentale de l’Université Laval/LOEX, Quebec City, QC G1J 1Z4, Canada; 5 UMR-CBMN, CNRS-Université de Bordeaux-IPB, 33600 Pessac, France

**Keywords:** extracellular vesicles, metastatic uveal melanoma, hepatic stellate cells, endothelial cells

## Abstract

Uveal melanoma (UM) is the most common primary intraocular tumor and often spreads to the liver. Intercellular communication though extracellular vesicles (EVs) plays an important role in several oncogenic processes, including metastasis, therapeutic resistance, and immune escape. This study examines how EVs released by UM cells modify stellate and endothelial cells in the tumor microenvironment. The surface markers, and the concentration and size of EVs derived from UM cells or choroidal melanocytes were characterized by high-resolution flow cytometry, electron microscopy, and Western blotting. The selective biodistribution of EVs was studied in mice by fluorescence imaging. The activation/contractility of stellate cells and the tubular organization of endothelial cells after exposure to melanomic EVs were determined by traction force microscopy, collagen gel contraction, or endothelial tube formation assays. We showed that large EVs from UM cells and healthy melanocytes are heterogenous in size, as well as their expression of phosphatidylserine, tetraspanins, and Tsg101. Melanomic EVs mainly accumulated in the liver and lungs of mice. Hepatic stellate cells with internalized melanomic EVs had increased contractility, whereas EV-treated endothelial cells developed more capillary-like networks. Our study demonstrates that the transfer of EVs from UM cells leads to a pro-fibrotic and pro-angiogenic phenotype in hepatic stellate and endothelial cells.

## 1. Introduction

Uveal melanoma (UM) is the most common primary intraocular tumor in adults, and occurs when melanocytes from the choroid, ciliary body, or iris transform into melanoma cells [1]. Despite the successful treatment of the eye tumor, half of all cases develop incurable liver metastases within 15 years, and long-term survival is uncommon [2,3]. Metastatic UM is notoriously recalcitrant to available oncology treatments [4,5]. The liver is the first and most common site of metastasis in 90% of UM cases, and subclinical/dormant micrometastatic foci are present at the time of the eye tumor diagnosis [3,6,7,8]. The molecular basis of this hepatotropism is not fully understood, but some tumors succeed in creating a pre-metastatic niche by manipulating the liver microenvironment to render it more hospitable to metastatic growth before the malignant cells even colonize this organ [9,10].

Accumulating evidence suggests that tumor-derived extracellular vesicles (EVs) could be mediators of this organotropism [11], and act as “seeds” in the crosstalk with a specific organ microenvironment (the “soil”), as proposed by Paget’s “seed and soil” theory [12]. EVs are broadly classified into two main types according to their biogenesis: the nm-sized small EVs (formerly called exosomes) that are secreted by cells via exocytosis of multivesicular endosomes, and the sub-µm large EVs (formerly called microvesicles) which are shed from the plasma membrane [13,14]. Recently, another large EV population unique to cancer has been identified. Known as large oncosomes (LOs), these EVs are 1–10 µm in diameter and originate from highly migratory tumor cells [15,16,17]. EVs selectively capture a repertoire of biological molecules such as proteins, nucleic acids, lipids, and metabolites [13], and shuttle these functional molecules within the tumor microenvironment and to distant metastatic sites [18,19]. They have surface molecules that enable tissue- or cell-specific targeting, which, upon reaching their recipient cells, can induce signaling in target cells via receptor–ligand interaction, or be internalized to deliver their contents [20]. To date, few studies have characterized or examined the role EVs play in UM [11,21,22,23,24,25,26,27,28,29].

Hepatic stellate cells (HSteCs) are localized in the perisinusoidal space (between the sinusoids and hepatocytes) and are particularly interesting, since they orchestrate liver fibrogenesis and antitumor immune response, thus creating a favorable microenvironment for metastatic cancers [30]. We have previously demonstrated that activated HSteCs synthesized fibrillar collagen in the vicinity of metastases in both UM patient samples and xenografted mice [31]. The co-inoculation of human HSteCs with UM cells also provided a permissive microenvironment that increased the number of hepatic micro-metastatic foci in immunodeficient mice [31]. In addition, the secretome of UM cells contains various proteins involved in stellate cell activation [32,33], with the majority of those secreted by exosomal mechanisms [32]. These findings warranted further investigation of EV-mediated intercellular communication between UM cells and liver cells and the role EVs play in the formation of a pre-metastatic niche.

EVs play an active part in the dissemination and the engraftment of metastatic cells by increasing endothelial permeability and neoangiogenesis, and by reprogramming stromal cells, which modify the extracellular matrix composition and architecture [18,19,34,35,36,37]. We hypothesized that tumor-derived EVs may influence the vasculature and prime the liver for metastatic growth by activating stellate cells in the pre-metastatic niche, leading to pro-fibrogenic properties. Therefore, we isolated EVs from the culture supernatant of UM cells and choroidal melanocytes, and studied their functional impact on endothelial and stellate cells. We found that metastatic UM cells release more EVs than non-metastatic UM cells and choroidal melanocytes. The uptake of melanomic EVs by stellate cells increased their activation and contractility, whereas in endothelial cells, they had pro-angiogenic properties. In addition, melanomic EVs accumulated in the liver of immunodeficient mice compared to EVs derived from non-melanomic cells. Our study shows direct evidence of intercellular communication through EVs between UM cells and stellate/endothelial cells.

## 2. Materials and Methods

### 2.1. Cell Culture

This study followed the tenets of the Declaration of Helsinki and was approved by our institutional human research ethics committee (Centre de recherche du CHU de Québec-Université Laval, Quebec City, Canada; protocols #2016-2529 and #2021-5273). The non-metastatic UM cell line Mµ2 (CRB CelluloNet, Lyon, France) and metastatic UM cell lines Mµ2F (CRB CelluloNet, Lyon, France) and TJU-UM001 (kindly provided by Dr. Takami Sato, Thomas Jefferson University, Philadelphia, PA, USA) were derived from primary ocular tumors or liver metastases, and were grown as previously described [38,39]. The hTERT-HSC cell line (human HSteCs; kindly provided by Dr. David A. Brenner, University of California San Diego, San Diego, CA, USA) was cultured as previously described [40]. GFP-HUVECs (human umbilical vascular endothelial cells; kindly provided by Dr. François A. Auger, Université Laval, Quebec City, Canada) were cultivated in EGM-2 medium with supplements (Lonza). The HEK293T cell line (ATCC #CRL-3216) was grown according to the recommendations of ATCC. Normal choroidal melanocytes (NCMs) were isolated from human eyeballs (Centre Universitaire d’Ophtalmologie’s Eye Bank, Quebec City, QC, Canada) after the written informed consent for research purposes was obtained from the donor’s next-of-kin [41,42]. NCMs were grown using melanocyte complete medium and FNC Coating Mix (AthenaES) as previously described [42], and the purity of the cultures was confirmed by immunostaining (mouse anti-human Melan-A antibody, clone A103, dilution 1:50; Agilent Dako). All cell types were grown at 37 °C in a humidified atmosphere with 5% CO_2_ and were tested routinely for mycoplasma infection by PCR (Universal Mycoplasma Detection Kit, ATCC).

### 2.2. Extracellular Vesicle Isolation

EV preparations were isolated and characterized according to the guidelines of the International Society for Extracellular Vesicles (ISEV) [43]. One million cells were plated in a 10 cm dish and grown to 70% confluence. UM cells, NCMs, HSteCs, or HEK293T cells were then cultivated for 48 h with EV-depleted culture media (centrifuged at 100,000× *g* for 2 h using a Beckmann Coulter OPTIMA L-90K with a 70 Ti rotor). The conditioned culture medium (10 mL per 10 cm dish) was centrifuged at 300× *g* for 10 min to remove floating cells, then at 2000× *g* for 20 min to remove dead cells and debris (Sorvall Legend RT Plus). The supernatant was collected and ultracentrifuged at 21,000× *g* for 90 min (Beckmann Coulter OPTIMA L-90K with a 70 Ti rotor) to recover the large EVs. Throughout this study, we will use the term “large EVs” to define the subpopulation of vesicles that is enriched in the 21,000× *g* centrifuge fraction. The supernatant was discarded and large EVs were diluted with 100 µL of HEPES-buffered saline (HBS: 150 mM NaCl, 2 mM CaCl_2_, 10 mM HEPES, pH 7.4) and quantified (particles/mL and µg of total proteins); then, aliquots (100 µg) were stored at −80 °C until their use.

### 2.3. High-Resolution Flow Cytometry and Nanoparticle Tracking Analysis

The concentration and size distribution of EVs were measured by high-resolution flow cytometry (HR-FC) using a BD FACSCanto II combined with a forward scatter (FSC) coupled to a photomultiplier tube (PMT) “small particles option” (CHU de Québec-Université Laval flow cytometry platform) [44,45]. EV samples were labeled for 30 min at room temperature with the Alexa Fluor^®^ 647 mouse anti-human CD63 tetraspanin antibody (clone H5C6, dilution 1:10; BD Biosciences), the BV421 Annexin V (which binds to phosphatidylserine (PS); dilution 1:50; BD Biosciences), and the CellTrackerTM Green CMFDA Dye (dilution 1:50; Invitrogen) diluted in Annexin V Binding Buffer (BD Biosciences). CMFDA is a non-fluorescent probe that freely passes through the plasma membrane and is converted to a fluorescent impermeant molecule by intracellular esterases in intact EVs. To confirm our CMFDA-positive events were EVs, control samples were treated with a detergent (0.05% Triton X-100) which dissolved the EV membrane moiety [46]. Antibody-alone samples were used in all experiments to control for antibody aggregates and background fluorescence. EV size gates were previously determined by our core cytometry facility using 100 nm, 500 nm, and 1000 nm silica beads (Kisker Biotech Germany). Particles between 100–1000 nm and that were CMFDA-positive were considered EVs. To accurately determine the concentration of EVs for each cell type, a known quantity of 2 µm Cy-5 labeled silica beads (Nanocs) was added to each sample at the same final concentration. For each sample, the same amount of Cy-5 labeled silica beads were collected and the total acquired volume (mL) of sample was calculated. The total count of each EV subpopulation was normalized to the volume of sample acquired (particles/mL). Data were analyzed using FlowJo software (version X.10.07r2; BD). The concentration and size distribution of EVs were also determined by Nanoparticle Tracking Analysis (NTA; scatter mode) using a NanoSight NS300 (Malvern Panalytical; Centre d’optique photonique et laser-Université Laval). EV samples were diluted to provide counts within the linear range of the instrument. Three videos of 1-min duration were recorded per sample, with a frame rate of 30 frames per second. Particle movement was analyzed by NanoSight NTA software v2.3 (Malvern Panalytical).

### 2.4. Immuno-Gold Labeling and Cryo-Transmission Electron Microscopy (Cryo-TEM)

Gold nanoparticles (NPs) were conjugated with Annexin-5 (Anx5; which binds to PS) or with mouse anti-human CD63 primary antibody (clone H5C6; BD Biosciences) as previously described [47,48]. During EV formation, phospholipid rearrangement at the plasma membrane leads to the externalization of PS on released EVs [49]. Consequently, PS is a common marker used to identify EVs. CD63 is a member of the tetraspanin family of membrane spanning molecules which have been shown to be enriched in EVs [43]. Since these are both common markers of EVs throughout the literature, we chose to characterize their expression in our large EVs produced by healthy liver cells and metastatic UM cells. EV samples were diluted 100x with the HBS buffer and labeled for 1 h with 1–4 × 10^15^ gold-NPs/L of 10 nm anti-CD63-gold-NP, or for 30 min with 1–4 × 10^15^ gold-NPs/L of 10 nm Anx5-gold-NP. Immuno-gold labeled EV samples were then processed for cryo-TEM [47]. A 4 µL aliquot was deposited on an EM grid coated with a perforated carbon film, and then the liquid was blotted from the backside of the grid, which was quickly plunged into liquid ethane using a Leica EMCPC cryo-chamber. EM grids were stored in liquid nitrogen prior to EM observation. Cryo-TEM was performed with a Tecnai F20 microscope (FEI) equipped with an Eagle 2k CCD camera (Thermo Fisher Scientific, Waltham, MA, USA). The percentage of CD63-positive or PS-positive EVs was determined by counting a mean of 120 or 150 EVs per condition, respectively.

### 2.5. Western Blotting

Cell pellets or EVs were lysed on ice in radioimmunoprecipitation assay (RIPA) buffer (50 mM Tris-HCl, pH 7.4, 150 mM NaCl, 0.6% NP-40) containing protease and phosphatase inhibitors. Lysates were then cleared of cellular debris by a 16,000× *g* centrifugation step for 10 min at 4 °C. Proteins were quantified by a BCA Assay (Pierce) and denatured with a 2X sample buffer (62.5 mM Tris-HCl, pH 6.8, 2% SDS, 25% (*v*/*v*) glycerol, 0.01% bromophenol blue, 5% β-mercaptoethanol). Samples (20 µg) were blotted onto nitrocellulose membranes (Bio-Rad Laboratories, Hercules, CA, USA), and the Ponceau S stain was used to detect the total proteins and confirm equal loading/transfer for both cellular and extravesicular samples. Membranes were blocked for 1 h at room temperature with phosphate-buffered saline (PBS) 1X-5% non-fat milk-0.1% Tween^®^ 20 was then incubated overnight at 4 °C with the following primary antibodies: mouse anti-α-tubulin antibody (clone DM1A, dilution 1:2500; Abcam), mouse anti-human CD9 tetraspanin antibody (clone HI9a, dilution 1:1000; BioLegend), mouse anti-human CD63 tetraspanin antibody (clone H5C6, dilution 1:1000; BioLegend), mouse anti-human CD81 tetraspanin antibody (clone 5A6, dilution 1:1000; Abcam), mouse anti-human Melan-A antibody (clone A103, dilution 1:1000; Agilent Dako), rabbit anti-tyrosinase related protein 1 (TYRP1; H-90) antibody (dilution 1:1000; Santa Cruz Biotechnology), or mouse anti-Tsg 101 antibody (clone C-2, dilution 1:1000; Santa Cruz Biotechnology). Membranes were washed twice in PBS 1X-0.1% Tween^®^ 20 and then incubated with anti-mouse or anti-rabbit HRP-conjugated secondary antibodies for 1 h at room temperature (dilution 1:2500; Jackson ImmunoResearch Laboratories). Membranes were washed twice in PBS 1X-0.1% Tween^®^ 20, followed by one wash with PBS, before using the WesternSure™ PREMIUM Chemiluminescent Substrate (LI-COR). The chemiluminescence was visualized using the C-DiGit Blot Scanner (LI-COR) and Image StudioTM Lite software (LI-COR).

### 2.6. Biodistribution of EVs

All animal experiments were conducted in voluntary compliance with the ARVO Statement for the Use of Animals in Ophthalmic and Vision Research and were approved by our institutional animal experimentation committee (Université Laval, protocol #2019-113). Large EVs (100 µg) derived from UM cells, HSteCs, or HEK293T cells (negative control of hepatotropism [50]) were labeled with the ExoGlowTM-Membrane EV Labeling Kit (System Biosciences) and injected into NOD CRISPR Prkdc Il2r Gamma (NCG) triple-immunodeficient mice (Charles River Laboratories) via the retro-orbital sinus (negative control: EV-depleted PBS). Mice were euthanized after 24 h, and the presence of ExoGlow-labeled EVs in various organs (liver, spleen, lungs, heart, kidneys) was assessed by fluorescence using the IVIS Lumina II and Living Image^®^ software (version 4.3; PerkinElmer, Waltham, MA, USA).

### 2.7. EV Internalization

Large EVs (100 µg) isolated from NCMs or UM cells were labeled with the VybrantTM DiI Cell-Labeling Solution (Invitrogen) according to the manufacturer’s instructions, and added to HSteCs for 24 h. As controls, HSteCs were pretreated for 24 h with 80 µM Dynasore (Cayman Chemical) to block caveolae-mediated endocytosis, or EVs were pretreated for 30 min with 100 µg/mL proteinase K (Invitrogen) to reduce their interaction with the membrane receptors present on HSteCs [51,52]. For the immunofluorescence analysis, HSteCs cultured on coverslips were washed with PBS, fixed with 4% paraformaldehyde in PBS for 10 min, and permeabilized using 0.1% Triton X-100 for 2 min. Fixed cells were blocked with PBS 1X-2% BSA for 5 min and incubated with a mouse anti-smooth muscle actin primary antibody (αSMA, marker of activated HSteCs [53]; clone 1A4, dilution 1:100; Agilent Dako) and the Phalloidin-iFluor™ 647 Conjugate (which labels actin filaments (F-actin) [54]; dilution 1:250; Cayman Chemical) for 1 h at room temperature. Next, cells were washed three times in PBS, and incubated with an anti-mouse Alexa 488 secondary antibody for 1 h at room temperature in the dark (dilution 1:250; Invitrogen). Samples were then washed three times in PBS (the second wash contained DAPI, dilution 1:5000; Invitrogen), rinsed in water, and finally mounted on slides using Fluoromount G (Electron Microscopy Sciences). Slides were observed using a LSM 800 confocal microscope (Carl Zeiss Canada), and images were processed with Zen Blue software (version 2.3; Carl Zeiss Canada). For the flow cytometry analysis, HSteCs exposed to DiI-labeled EVs for 24 h were trypsinized and resuspended in PBS 1X, and then analyzed with a BD Accuri C6 Plus flow cytometer. The events were plotted as 2D-dot plots with FlowJo software (version X.10.07r2; BD) using the forward scatter (FSC) and FL2 height signals to determine the percentage of HSteCs that had internalized fluorescent EVs.

### 2.8. Traction Force Microscopy

Polyacrylamide gels containing 0.5 μm diameter fluorescent beads (Life Technologies, Carlsbad, CA, USA) were prepared with a Young’s moduli of 5 kPa using a ratio 7.5:0.175 of acrylamide (40% *w*/*v*; Bio-Rad) to bis-acrylamide (2% *w*/*v*; Bio-Rad) and then coated with 0.1 mg/mL of rat tail type I collagen (Corning) [55,56]. HSteCs (20,000 cells) were grown in 35-mm dishes for 24 h, and then large EVs from TJU-UM001 cells (100 µg) were deposited onto adherent stellate cells. After 24 h, phase contrast images of single HSteCs and fluorescent images of the bead field at the surface of the polyacrylamide gel were acquired (Nikon Eclipse Ti microscope, NIS-Elements software) [56,57]. Cells were removed from the polyacrylamide gel using 0.25% trypsin/EDTA, and a fluorescent image of the bead field was acquired after cell removal [56,57]. Bead displacements between stressed (+EVs) and null (w/o EVs) states were analyzed for 25 cells per condition using the LIBTRC library to obtain traction stress maps (Pa) and traction force values (nN) [56,58].

### 2.9. Collagen Gel Contraction Assay

The type I collagen was isolated from rat tail tendons and solubilized in 0.1% sterile acetic acid to get a 10 mg/mL stock solution. The collagen stock solution was then diluted to 1 mg/mL with 0.1% acetic acid, incubated for 5 days at 4 °C, and neutralized with PBS 10X, 250 mM HEPES, 440 mM NaHCO_3_ to form a collagen solution, followed by the addition of 1 M NaOH (4 µL/mL). Next, a 24-well plate was pre-coated with BSA for 1 h at room temperature to avoid gel attachment to the bottom of the plate. Collagen gels were mixed with HSteCs (pre-exposed or not for 24 h to large EVs (100 µg) derived from NCMs or UM cells) and added to the 24-well plate (40,000 cells per well in 500 µL of collagen). After 1 h of incubation at 37 °C, the outward edge of the gel was carefully detached with a pipette tip, and phase contrast pictures were taken at 0 h, 24 h, and 48 h using a stereomicroscope (Zeiss SteREO Discovery v8 with an AxioCam ERc5s, Axio Vision software version 4.8). The collagen gel area was quantified (in percentage) using the ImageJ software (version 1.50; https://imagej.nih.gov/ij/index.html; accessed date: 1 August 2022).

### 2.10. Endothelial Tube Formation Assay

GFP-HUVECs were seeded at 200,000 cells per well in a 6-well plate and grown for 48 h. Large EVs (100 µg) isolated from UM cells were labeled with the VybrantTM DiI Cell-Labeling Solution (Invitrogen) for 30 min at 37 °C in the dark. DiI-labeled EVs were purified with PD SpinTrap G-25 columns (Cytiva) following the manufacturer’ protocol. As controls, GFP-HUVECs were pretreated for 24 h with 80 µM Dynasore (Cayman Chemical), or EVs were pretreated for 30 min with 100 µg/mL proteinase K (Invitrogen). At 24 h, large EVs were added to the 6-well plate to be internalized by GFP-HUVECs during 24 h. At 48 h, Matrigel^®^ layers were deposited into wells of concavity slides (Electron Microscopy Sciences) and incubated for 1 h at 37 °C. GFP-HUVECs (30,000 cells per well in 100 µL) were added onto the Matrigel^®^ layers and incubated at 37 °C in culture medium with or without angiogenic supplements to perform the endothelial tube formation assay [59]. Phase contrast images were taken at 2 h, 4 h, 6 h, and 12 h, whereas representative fluorescence images were taken at 6 h. Then, samples were fixed with 4% paraformaldehyde in PBS for 10 min and stained with DAPI (dilution 1:5000; Invitrogen). The characteristics of the pseudo vascular organization of GFP-HUVECs were analyzed using the Angiogenesis Analyzer for ImageJ.

### 2.11. Statistical Analysis

Data were reported as the mean ± standard error (SEM) of three or more experiments (*n* > 3) unless stated otherwise. Statistical significance (*p* < 0.05 or less) was determined with Prism 9 (GraphPad Software, San Diego, CA, USA) using two-way analysis of variance (ANOVA) with Sidak’s multiple comparisons test unless stated otherwise.

## 3. Results

### 3.1. Greater Release of Large EVs by UM Cells Compared to Normal Melanocytes

**Figure 1:**
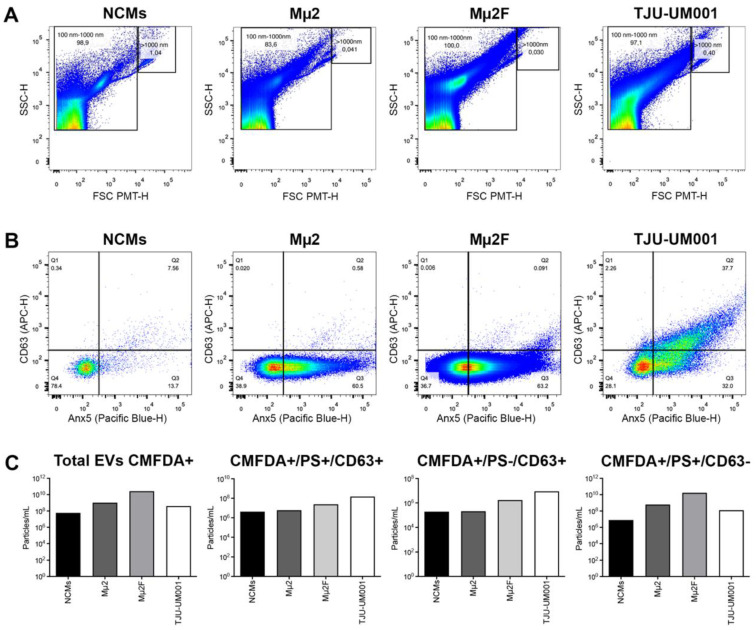
UM cells release more EVs than melanocytes. The size distribution of EVs derived from NCMs, non-metastatic UM cell line Mµ2, and metastatic cell lines Mµ2F and TJU-UM001 was determined by HR-FC. (**A**) EV size was determined with forward and side scatter, and EVs between 100–1000 nm were included in the analysis. (**B**) The NCM- and UM-derived EVs were stained with CMFDA to identify intact EVs and exclude debris. CMFDA-positive events were analyzed for Anx5 (PS)/CD63 expression. (**C**) The concentration of EVs (particles/mL) for the various CMFDA+/PS/CD63 subpopulations was determined by normalizing the number of particles to the total volume acquired for each sample.

**Figure 2:**
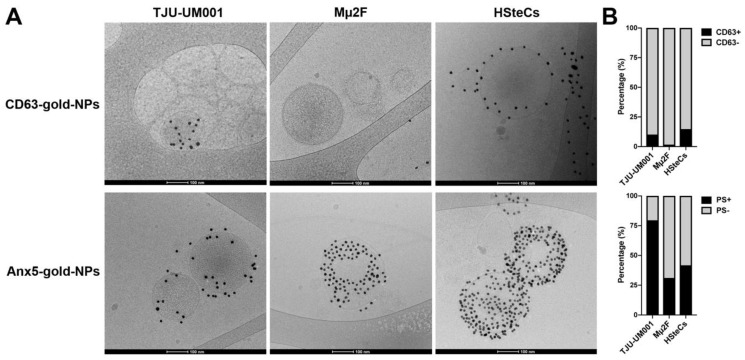
Morphology of UM-derived EVs. (**A**) Large EVs isolated from metastatic UM cell lines TJU-UM001 and Mµ2F or HSteCs (healthy liver cell controls) were labeled with 10-nm CD63-gold-NPs or Anx5-gold-NPs (dark particles) for cryo-TEM analysis. Scale bars, 100 nm. (**B**) The percentage of EVs positive for CD63 or PS was quantified for each cell type.

Since there is considerable heterogeneity within secreted EVs from different cell types (either EV populations according to size or particle subpopulations according to surface markers and luminal content [60]), which adds another layer of complexity that remains to be addressed in UM, we first wanted to characterize the particles produced by non-metastatic (Mµ2) and metastatic (TJU-UM001, Mµ2F) UM cell lines or NCMs (healthy control) after their isolation by differential centrifugation. We thus analyzed the size distribution, concentration, and presence of well-known EV markers (CD63 and PS [43]) of the large EVs using HR-FC (Figure 1), cryo-TEM (Figure 2), or NTA (Table 1).

We first determined the size range of EVs by examining the size of all the events as determined by SSC/FSC-PMT (Figure 1A). For all cell types, most events were between 100 and 1000 nm in diameter (98.9%, 83.6%, 100%, 97.1%, respectively for NCMs, Mµ2, Mµ2F, and TJU-UM001), consistent with the expected size of large EVs. We then labeled the EVs with CMFDA, Anx5 (PS), and the tetraspanin CD63. CMFDA-positive events were considered intact EVs, and the expression of PS and CD63 was examined for these events (Figure 1B). The concentration of CMFDA+/PS+/CD63+, CMFDA+/PS−/CD63+, and CMFDA+/PS+/CD63− EVs was determined by normalizing the total events in each subpopulation to the volume of sample analyzed (particles/mL) (Figure 1C). The CMFDA dye allowed us to confirm the presence of intact EVs following our ultracentrifugation isolation procedure. The concentration of total intact EVs was higher in the UM cell lines compared to the non-cancerous NCMs (18, 462, and 7 times higher for Mµ2, Mµ2F, and TJU-UM001, respectively). The concentration of EVs positive for both PS/CD63 was increased in the metastatic UM cell lines compared to the non-cancerous NCMs (6 and 34 times higher for Mµ2F and TJU-UM001, respectively).

In addition, the metastatic UM cells released more EVs positive for both markers than the non-metastatic UM cells (4 and 25 times higher for Mµ2F and TJU-UM001, respectively) (Figure 1C). Similarly, the concentration of large EVs PS−/CD63+ was higher in the metastatic UM cell lines compared to the NCMs (9 and 46 times higher for Mµ2F and TJU-UM001, respectively) or the non-metastatic UM cells (8 and 42 times higher for Mµ2F and TJU-UM001, respectively). The UM cells released more EVs PS+/CD63− than the NCMs (79, 2125, and 16 times higher for Mµ2, Mµ2F, and TJU-UM001, respectively), whereas their concentration was higher in the metastatic UM cell line Mµ2F compared to its non-metastatic counterpart (27 times higher). PS was more present at the surface of large EVs than CD63 for all cell types (PS: 21–70% of EVs; CD63: 0.1–40% of EVs). Only 22% of intact EVs derived from melanocytes were positive for PS and/or CD63 compared to 61–72% for UM cells.

The mean size and concentration of particles were also determined by NTA using the scatter mode (Table 1). EVs derived from metastatic UM cells were bigger in size than those isolated from non-metastatic UM cells and melanocytes (mean size: 213, 200, 180, and 96 nm for TJU-UM001, Mµ2F, Mµ2, and NCMs, respectively). The total events detected by NTA exceeded flow cytometric EV counts by more than one or two orders of magnitude for most samples. This is in agreement with the EV characterization literature [61], since NTA in scatter mode is unable to discriminate intact EVs from other light-scattering entities lacking a lipid membrane, such as protein aggregates or lipoproteins. The concentration of particles was still higher in UM cell lines compared to melanocytes (2 and 3 times higher for Mµ2 and TJU-UM001, respectively).

Next, large EVs derived from metastatic cell lines Mµ2F/TJU-UM001 and from melanocytes or stellate cells (healthy controls) were visualized by cryo-TEM using gold NPs conjugated to anti-CD63 or Anx5 (PS) (Figure 2). The experiment was originally performed using NCMs as healthy controls; however, there were not enough CD63/PS positive EVs recorded in the microscopic fields to perform an accurate quantification. This is consistent with our data showing that healthy melanocytes produce significantly less EVs than tumor cells. As seen in Figure 2A, spherical EVs ranging mostly from 100 to 500 nm in diameter were covered with Anx5-gold-NPs or anti-CD63-gold-NPs. Since not all EVs were positive for these markers, we quantified the percentage of CD63+ or PS+ EVs in our samples (Figure 2B). Large EVs displayed more PS at their surface (79.5%, 31.1%, and 41.7% for TJU-UM001, Mµ2F, and HSteCs, respectively) than CD63 (10.1%, 1.6%, and 14.8% for TJU-UM001, Mµ2F and HSteCs, respectively). These results illustrate the heterogeneity of EV size and CD63/Anx5 labeling in UM cells and NCMs and demonstrate a significant increase of large EV release as a function of cancer progression. This suggests that the luminal content of EVs is also most likely altered according to UM stages.

### 3.2. Presence of EV Protein Markers in Melanomic and Melanocytic EV Fractions

**Figure 3:**
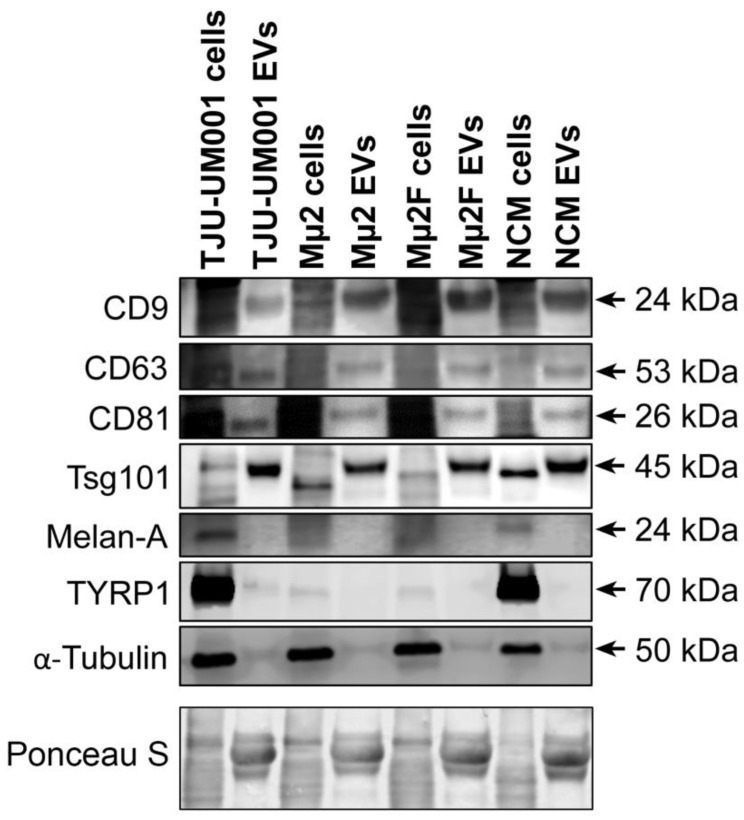
Exosomal and melanocytic markers present in UM-derived EVs. Cell pellets and EV fractions isolated from UM cell lines TJU-UM001 (metastatic), Mµ2 (non-metastatic), and Mµ2F (metastatic), or NCMs, were analyzed for their expression of exosomal (CD9, CD63, CD81, Tsg101) or melanocytic (Melan-A, TYRP1) markers by Western blotting. The anti-α-tubulin was used as marker of cytosolic proteins. Ponceau S staining was used as a loading control. Arrows indicate the specific band for each marker.

Given that our current results indicate that the UM progression can impact the molecular characteristics of the EV surfaceome and cargo, we extended our analysis to a panel of well-known EV markers (surface tetraspanins CD9/CD63/CD81 and Tsg101 [43]) and proteins specific to the melanocytic lineage (Melan-A and TYRP1 [62,63]). According to immunoblotting (Figure 3), EV fractions collected from UM cell lines TJU-UM001, Mµ2, and Mµ2F, or from melanocytes, were all enriched in tetraspanins CD9, CD63, and CD81. Tsg101, a component of the ESCRT-1 complex and an important regulator of the vesicular trafficking process [20], was also enriched in large EVs of all cell types. Next, Melan-A and TYRP1 melanocytic proteins were barely detectable in EV fractions compared to the cell pellets. Taken together, these results show that large EVs released by UM cells and melanocytes expressed various EV markers, whereas the tested melanocytic markers were not abundant in EV fractions. Therefore, Melan-A and TYRP1 are not good candidates as markers of melanomic EVs, and the search for such a marker is still ongoing.

### 3.3. Accumulation of Melanomic EVs in Lungs and Liver of Immunodeficient Mice

**Figure 4:**
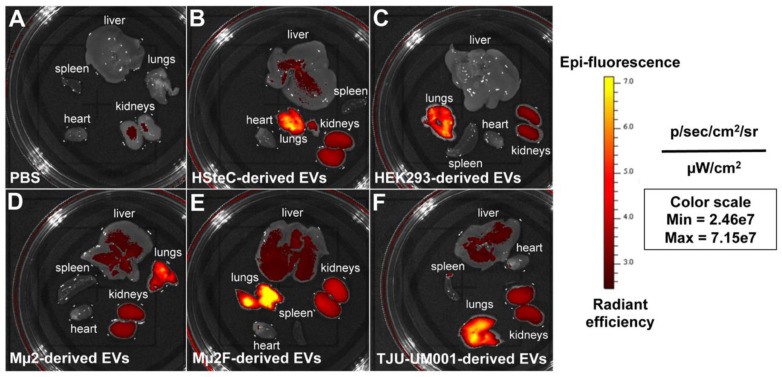
Accumulation of melanomic EVs in the liver and lungs of immunodeficient mice. (**A**) Inoculation of PBS served as control without EVs. (**B**) Large EVs derived from HSteCs, (**C**) HEK293, or (**D**–**F**) UM cell lines (Mµ2, Mµ2F, TJU-UM001) were labeled with Exoglow before being inoculated in the retro-orbital sinus of NCG mice (*n* = 4/condition). Fluorescence of the liver, lungs, kidneys, heart, and spleen was analyzed with the IVIS after 24 h.

Since it has been previously reported that tumoral EVs are involved in metastatic organotropism [11], we examined the biodistribution of fluorescent large EVs isolated from UM cell lines Mµ2, Mµ2F, and TJU-UM001 following injection into the retro-orbital sinus of NCG triple-immunodeficient mice (Figure 4). Interestingly, melanomic and HSteC-derived EVs accumulated in the liver, whereas HEK293 EVs (negative control of hepatotropism [50]) did not. EVs of all origins accumulated strongly in the lungs at 24 h, which is consistent with previous studies that show EVs initially reach the lungs before redistributing to other organs [64]. A fluorescent signal was also detected in kidneys for all EV samples, which could be associated with clearance of EVs. No signal was found in the heart or spleen. Taken together, these results demonstrate that melanomic EVs specifically target the liver, thus reflecting their hepatotropism.

### 3.4. Activation of Hepatic Stellate Cells by UM-Derived EVs

**Figure 5:**
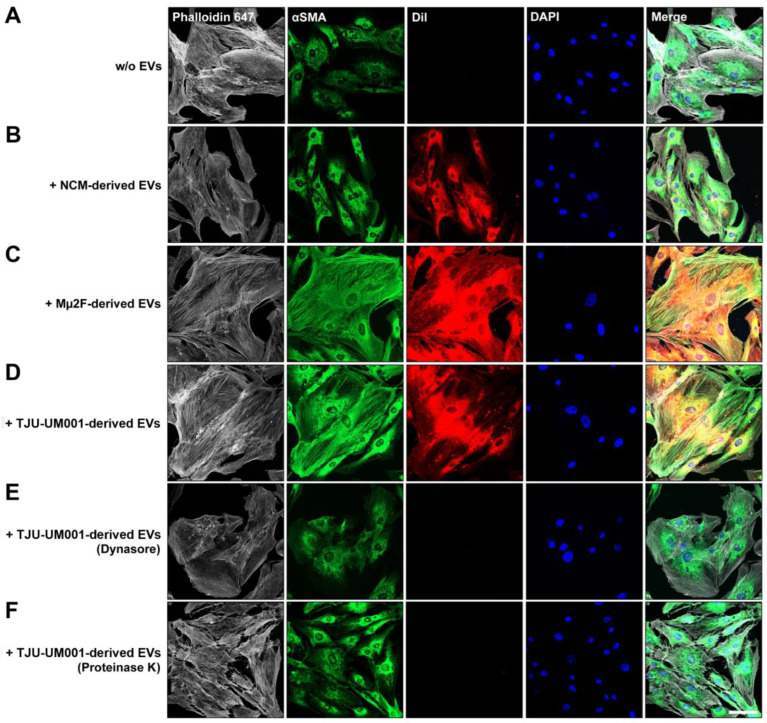
Activation of stellate cells after the internalization of UM-derived EVs. (**A**) The expression of F-actin (Phalloidin 647; grey) and α-SMA (green) was assessed in HSteCs without addition of EVs by confocal microscopy. Nuclei were counterstained with DAPI (blue). (**B**) Large EVs isolated from NCMs (normal cell control) or (**C**) metastatic UM cell lines Mµ2F and (**D**–**F**) TJU-UM001 were labeled with DiI (red) and added to HSteCs for 24 h to allow for their uptake before observing the expression of F-actin (grey) and α-SMA (green). (**E**) HSteCs were pretreated with Dynasore, or (**F**) TJU-UM001-EVs were pretreated with proteinase as controls. Scale bar, 50 µm.

**Figure A1:**
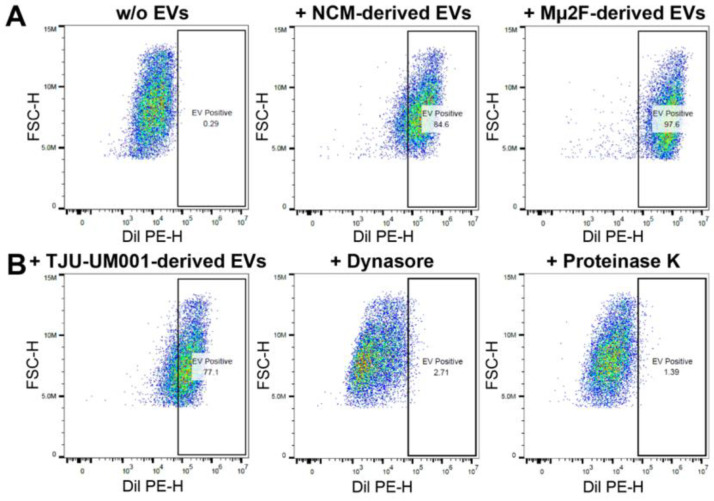
Dynasore and proteinase K pretreatments prevented EV internalization in stellate cells. (**A**) Fluorescence of HSteCs without addition of EVs by flow cytometry. Fluorescence of HSteCs after the addition of DiI-labeled large EVs derived from UM cell lines (Mµ2F, TJU-UM001) or NCMs for 24 h (**A**,**B**). (**B**) Fluorescence of HSteCs pretreated with Dynasore or after the addition of TJU-UM001-EVs pretreated with proteinase K are shown as controls. The gates indicate the percentage of fluorescent HSteCs.

We have previously shown that a synergic interaction that promotes metastasis exists between UM and stellate cells [31]. It was demonstrated before that EVs shed by metastatic colorectal cancer cells can activate stellate cells [65], whereas breast tumor cell-derived EVs can activate fibroblasts and increase their contractility in conditions, mimicking the high matrix stiffness found in mammary tumors [56]. In this context, we wanted to determine if UM-derived EVs were able to activate stellate cells at an early stage and impact their matrix interactions before the invasion of the liver by UM cells and the concomitant remodeling of the hepatic extracellular matrix. To do so, HSteCs were incubated with DiI-labeled large EVs collected from metastatic UM cell lines (TJU-UM001 and Mµ2F) or non-cancerous NCMs. Then, immunostaining was performed to visualize F-actin and αSMA in cells that have internalized the EVs (Figure 5). First, the negative control without the addition of EVs showed that HSteCs grown on plastic had a basal expression of αSMA (Figure 5A). There was uptake of both melanomic (Figure 5C,D) and melanocytic (Figure 5B) EVs by stellate cells, as evidenced by the DiI red fluorescence. However, compared to the negative control, only UM-derived EVs increased the expression of αSMA and shifted its localization to the actin stress fibers (Figure 5C,D). Interestingly, pre-treatment of HSteCs with the dynamin inhibitor Dynasore, or pre-treatment of UM-derived EVs with proteinase K to digest surface proteins, completely suppressed the internalization of fluorescent EVs in stellate cells (Figure 5E,F and Figure A1), and prevented the expression of αSMA and its incorporation into actin stress fibers (Figure 5E,F).

**Figure 6:**
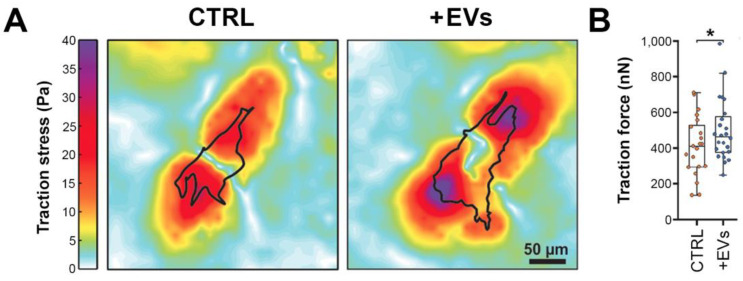
Increased contractility of stellate cells after the internalization of UM-derived EVs. HSteCs were grown on 5 kPa polyacrylamide gels containing fluorescent beads and exposed for 24 h to large EVs from metastatic UM cells TJU-UM001. Then, bead displacements between stressed (+EVs) and null (CTRL) states were measured using TFM. (**A**) Representative traction stress maps (Pa) and (**B**) traction force (nN) are shown for untreated (CTRL) or treated (+EVs) HSteCs. Scale bar, 50 µm. Plots are median ± the minimum and maximum values. * *p* < 0.05 (Welch’s *t*-test).

Next, we used TFM to measure the cellular contractile force of stellate cells following melanomic EV uptake using 5 kPa substrates that simulate higher matrix stiffness found in tumors (Figure 6). HSteCs that had internalized EVs produced by metastatic cells (TJU-UM001) showed higher traction stress (Figure 6A), indicating an increased ability to contract the extracellular matrix. Melanomic EVs also induced a significant increase in stellate cell traction force (median: 494 nN) compared to untreated cells (median: 413 nN, *p* < 0.05; Figure 6B). Overall, these results clearly demonstrate that the uptake of EVs by stellate cells is mediated through an active mechanism (as demonstrated by treatments with Dynasore or proteinase K). Interestingly, only melanomic EVs activated the stellate cells, which led to a myofibroblastic contractile phenotype.

### 3.5. Increase of Collagen Contraction by Hepatic Stellate Cells Treated with Melanomic EVs

**Figure 7:**
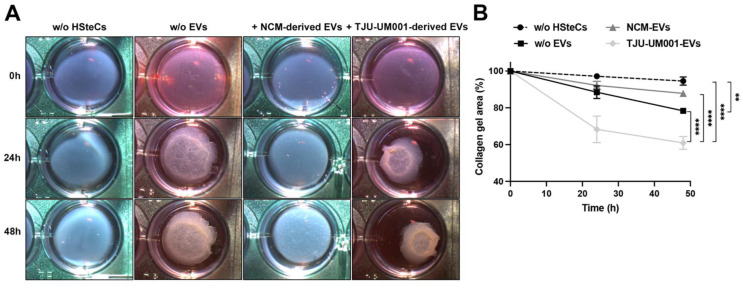
UM-derived EVs increase collagen gel contraction by stellate cells. (**A**) Representative images of collagen gels (1 mg/mL) without embedded HSteCs, with embedded HSteCs not treated with EVs, and with embedded HSteCs treated during 24 h with large EVs derived from NCMs or metastatic UM cell line TJU-UM001 taken with a stereomicroscope. (**B**) Corresponding quantification of the collagen gel area (in percentage) for each condition during 48 h. Error bars indicate ±SEM (*n* = 3); ** *p* < 0.01 and **** *p* < 0.0001 (two-way ANOVA with Sidak’s multiple comparisons test).

Since a desmoplasia was found near hepatic metastases in UM [31,66] and other cancers [67,68], we then evaluated if the internalization of melanomic EVs by HSteCs can lead to a change in their ability to remodel the extracellular matrix (Figure 7).

The contractile strength of stellate cells exposed to large EVs isolated from the metastatic cell line TJU-UM001 or from melanocytes was assessed using collagen gel contraction assays, in which HSteCs were embedded in type I collagen gels (Figure 7A). Collagen gels without cells or containing untreated HSteCs were included to monitor the basal contraction of the gel (Figure 7B; collagen gel area as percentage at 48 h: 95% vs. 78% (i.e., gel contraction of 5 vs. 22%, *p* < 0.01) for gel without cells and HSteCs w/o EVs). HsteCs exposed to UM-derived EVs exhibited a significant increase in their ability to contract collagen gels compared to untreated cells at 48 h post-seeding (Figure 7B; 78% vs. 61% (i.e., gel contraction of 22 vs. 39%, *p* < 0.0001) for HSteCs w/o EVs and HSteCs+TJU-UM001-derived EVs). Melanocytic EVs did not produce similar results (Figure 7B; 88% vs. 61% (i.e., gel contraction of 12 vs. 39%, *p* < 0.0001) for HSteCs+NCM-derived EVs and HSteCs+TJU-UM001-derived EVs), and they even decreased the contraction of the collagen gel in comparison to untreated HSteCs (Figure 7B; 78% vs. 88% (i.e., gel contraction of 22 vs. 12%, *p* > 0.05) for HSteCs w/o EVs and HSteCs+NCM-derived EVs). These results demonstrate that although EVs from both cell types were internalized by HSteCs, only UM-derived EVs had a functional impact on these stromal cells and changed their contractile function.

### 3.6. Increased Formation of Capillary-like Tubes in Presence of Melanomic EVs

**Figure 8:**
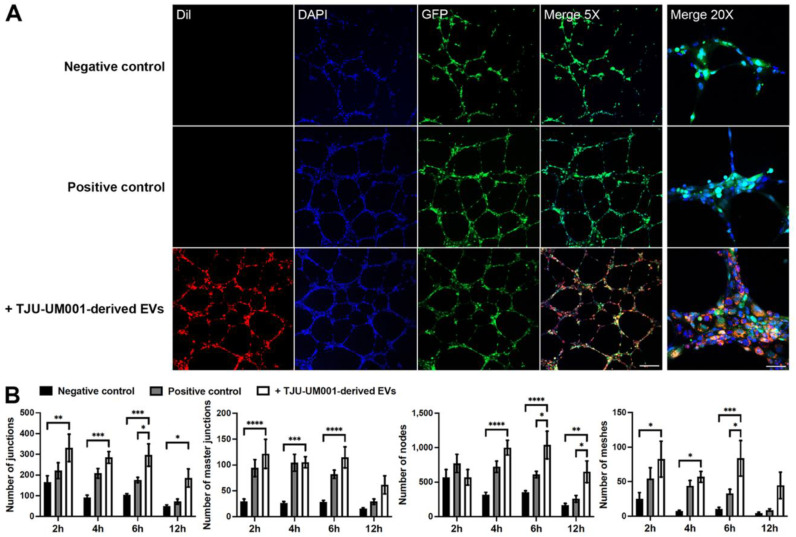
UM-derived EVs increased the formation of capillary-like tubes by endothelial cells. (**A**) Untreated GFP-HUVECs (green) were cultured for 12 h on Matrigel^®^ with medium without VEGF (negative control; top panels) or medium supplemented with VEGF (positive control; middle panels). Images are from the 6 h time point. Nuclei were counterstained with DAPI (blue). Scale bars: Merge 5X, 200 µm; Merge 20X, 50 µm. (**A**) Large EVs isolated from metastatic UM cell line TJU-UM001 were labeled with DiI (red) and added to GFP-HUVECs (green) for 24 h before their seeding on Matrigel^®^ for 6 h (bottom panels). (**B**) The number of junctions, master junctions, nodes, and meshes were quantified in function of time (hours) for each condition. Error bars indicate SEM (*n* = 3); * *p* < 0.05, ** *p* < 0.01, *** *p* < 0.001, and **** *p* < 0.0001 (two-way ANOVA with Sidak’s multiple comparisons test).

In addition to their effect on stromal cells, increasing evidence indicates that tumoral EVs can also have a pro-angiogenic function in cancer [69,70]. Thus, we hypothesized that melanomic EVs could also be involved in endothelial network formation. HUVEC-GFP were seeded on top of a Matrigel^®^ layer to promote tube formation mimicking capillaries (Figure 8).

**Figure A2:**
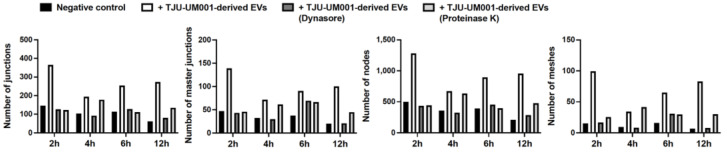
Dynasore and proteinase K pretreatments prevented UM-derived EV internalization in endothelial cells and inhibit the formation of capillary-like tubes. Untreated GFP-HUVECs were cultured for 12 h on Matrigel^®^ with medium without angiogenic factors (negative control; black bars). Large EVs isolated from metastatic UM cell line TJU-UM001 were added to GFP-HUVECs for 24 h before their seeding on Matrigel^®^ for 12 h (white bars). GFP-HUVECs were also pretreated with Dynasore (dark grey bars), or TJU-UM001-EVs were pretreated with proteinase K (light grey bars) as controls. The number of junctions, master junctions, nodes, and meshes were quantified in function of time (hours) for each condition (*n* = 1).

When DiI-labeled large EVs derived from the metastatic UM cell line TJU-UM001 were added to endothelial cells prior to their seeding on Matrigel^®^, the fluorescent red signal was found in the cytoplasm of GFP-HUVECs (Figure 8A). The internalization of melanomic EVs in GFP-HUVECs (in absence of angiogenic supplements in the culture medium) increased the number of capillary-like tubes and accelerated their formation in comparison to the negative control (culture medium w/o angiogenic supplements) (Figure 8A). The quantification of junctions, master junctions, nodes, and meshes in capillary-like tubes demonstrated that they were significantly augmented after the treatment with melanomic EVs (Figure 8B). The maximal effect on capillary-like tube formation was obtained 6 h after the beginning of the experiment (Figure 8A,B; 2.9 (junctions), 4.1 (master junctions), 2.9 (nodes), and 8.3 (meshes) times higher for HUVECs+TJU-UM001-derived EVs vs. negative control). These pro-angiogenic effects were inhibited by pre-treatment with Dynasore or proteinase K (Figure A2). These results show the role of UM-derived EVs in vascular network formation and stabilization, which could synergize with their pro-fibrotic action on stellate cells to promote metastasis.

## 4. Discussion

In recent years, EV-based clinical applications (e.g., predictive biomarkers and drug delivery vehicles) have gained attention in cancer due to EV biocompatibility and low immunogenicity/cytotoxicity, and because they play a role in tumor metastasis, drug resistance, and immune escape [64]. Here, we aimed to characterize EVs released in vitro by both UM cells and melanocytes to investigate their functional impact on hepatic stellate cells and endothelial cells. Our findings support the hypothesis that UM-derived EVs can prepare the future metastatic site for incoming tumor cells and support their engraftment and survival by acting as a “soil conditioner” during the formation of the pre-metastatic niche. Indeed, we showed a heterogeneity in EV size and surface protein markers in UM cells and found a significant increase of large EV release by metastatic UM cells compared to non-metastatic cells and choroidal melanocytes. We also demonstrated the hepatotropism of melanomic EVs, and their pro-fibrotic and pro-angiogenic activities on stellate/endothelial cells contrary to EVs derived from their normal counterpart.

To date, only a handful of studies have looked at EVs contained in UM patients’ biological fluids [21,22,23,26,29], or have been carried out with UM cell cultures or mouse models [11,24,25,27,28]. The majority of these studies analyzed small EVs (formerly called exosomes), whereas we focused on large EVs (enriched in the 21,000× *g* centrifuge fraction) for the presented data. Proteomics data from exosomes isolated from several cell lines revealed distinct integrin expression patterns between tumor models with a propensity to metastasize to specific sites (i.e., tropism) such as the brain, lung, and liver (such as UM), in which the exosomal integrin αvβ5 was linked to liver metastasis [11]. Recent studies have analyzed the proteome of EVs isolated from UM cell cultures using quantitative mass spectrometry [25,27,28]. These groups found that integrins were the most highly represented protein family [25,27,28], and confirmed the presence of the αv and β5 integrin subunits in EVs collected from primary UM cell lines (MP41, MP46, 92.1, Mel270, and Mel285). Interestingly, these proteins were not enriched in the EVs of the metastatic cell lines (OMM1.3 and OMM2.5) that were also tested [25,27,28]. These studies identified proteins involved in endocytosis, membrane trafficking, and metastasis in the cargo of UM-derived EVs [25,27,28]. Tsering et al. also looked at melanocytic markers and found that Melan-A, TYRP1, PMEL, melanotransferrin, or melanoma-associated antigens D1/D2 were detected in EVs of almost all UM cell lines [25]. In this study, we found that PS is more present in large EVs than tetraspanin CD63, but did not find an enrichment of the melanocytic markers Melan-A and TYRP1 in the extravesicular fractions. This demonstrates the heterogeneity within secreted EVs from different UM cell lines. Further studies are required to compare the molecular surfaceome/content, biodistribution, and biological activity of UM-derived small and large EVs [15,16,17]. LOs were previously found in prostate, lung, bladder, and brain cancer cell lines, and were associated with advanced disease [16,71,72,73,74,75], but their production by UM cells still needs to be confirmed.

We confirmed the hepatotropism of the UM-derived large EVs when injected in the retro-orbital sinus of immunodeficient mice. Interestingly, Ambrosini et al. demonstrated that a preconditioning of mice by inoculating UM-derived exosomes before injecting UM cells increased the development of hepatic metastases [28]. We were the first to explore the EV-based intercellular communication between UM cells and hepatic stellate cells or endothelial cells in vitro to better understand how it is promoting the pre-metastatic niche formation. Previous studies have shown that UM-derived EVs were internalized in hepatocytes in vitro [25,27,28]. The uptake of UM-derived exosomes by hepatocytes led to the release of prometastatic molecules such as MIF and CXCL1, an increase in fibronectin production, and the activation of cell signaling pathways such as MET, ERK, AKT, and STAT3, whereas the supernatant of hepatocytes exposed to melanomic exosomes increased the migratory capacity of UM cells [25,27,28]. Activated HSteCs and excessive collagen deposition were previously spotted surrounding UM micro- and macro-metastases in human samples and animal models [8,31,66,76,77,78,79]. This distinctive feature of the hepatic metastatic niche was exploited recently when a collagen I targeted protein-based contrast agent was tested in a preclinical mouse model for the early detection of UM liver metastases by magnetic resonance imaging [80]. We demonstrated in the present study that the uptake of melanomic EVs by stellate cells led to a myofibroblastic and highly contractile phenotype, and these activated cells exhibited a greater ability to contract 3D collagen gels compared to untreated or melanocytic EV-exposed stellate cells. It was previously shown that patients with a stiffer fibrotic liver have higher metastatic incidence and lower survival rate in colorectal cancer [81]. A stiff matrix can also impact the EV uptake by stromal cells, as previously demonstrated in breast cancer [56]. Thus, our data suggest that the increased extracellular matrix remodeling by stellate cells reprogrammed by melanomic EVs could promote the UM cell engraftment and growth in the liver, and that the concomitant change in matrix stiffness could be exploited in diagnostic imaging. Analyses of the composition and stiffness of the pathological extracellular matrix produced by activated HSteCs are ongoing with the tissues collected from our immunodeficient mice inoculated with UM-derived EVs.

Tumor vasculogenesis is critical in tumor growth and metastatic dissemination. Several studies have previously demonstrated that EVs are involved in neoangiogenesis and can increase vascular permeability to facilitate the formation of the pre-metastatic niche [34]. Exosomes from highly malignant cutaneous melanoma murine cells enhanced lung endothelial permeability in mice compared to exosomes from non-metastatic cells [82], whereas human cutaneous melanoma-derived exosomes induced pulmonary vascular leakage [11]. Breast tumor-derived EVs express a 90 kDa crosslinked form of VEGF (VEGF90) on their membrane that leads to a sustained activation of VEGF receptors on endothelial cells, and stimulated their migration and tubulogenesis. Interestingly, VEGF90 also has a weakened affinity for the therapeutic VEGF antibody bevacizumab [83]. The VEGF189 isoform was found at the surface of colorectal cancer cell-derived exosomes, which induced endothelial cell migration and tube formation independently of uptake, stimulated tumor xenograft growth, and was implicated in resistance to bevacizumab [84]. Colorectal cancer cell-derived exosomes were enriched with miR-25-3p, which promoted endothelial tube formation by HUVECs, disrupted the tight junctions of vascular endothelial cells, and allowed metastasis in murine liver and lungs [85]. In addition, proangiogenic proteins such as VEGF, IL-8, endothelin-1, and TGFβ1 were increased in hepatocytes treated with UM-derived exosomes [25,27,28]. In the present study, we demonstrated that UM-derived EVs increased the endothelial tube formation ability of HUVECs. This suggests that UM-derived EVs contain pro-angiogenic factors and play a role in vascular network formation and stabilization. The UM extravesicular content in angiogenic cytokines will need to be characterized in future work using more physiological target cells such as choroidal and hepatic endothelial cells to study the vascular permeability in presence of melanomic EVs at both the primary tumor and metastatic sites.

Finally, EV-mediated communication is a bi-directional process, as EVs released by activated stromal cells can also intensify the malignant behavior of tumor cells. We showed that HSteCs released EVs in vitro (Figure 2, Table 1); however, further experiments will be needed to study the feed-forward signaling loop mediated by activated stellate cell-derived EVs in the shaping of the ever-evolving metastatic microenvironment.

A potential limitation of this study is the fact that EVs from established cell lines (Mµ2, Mµ2F, and TJU-UM001) rather than patient-derived primary cultures of tumor biopsies (low number of passages) were used as models. Although primary cultures provide models closer to the in-situ microenvironment, working with these cell lines is technically challenging. Not only do you need access to fresh patient samples, but the cultivation and expansion of these cells in order to harvest the number of EVs required for repeated experiments is challenging. Another limitation is the use of monolayer culture models throughout these experiments. Although 2D models are an indispensable tool in molecular biology, 3D models, such as tumor spheroids, more accurately mimic the in-situ microenvironment with hypoxic areas and cellular heterogeneity. In future studies, we hope to use more complex 3D cell culture models (such as spheroids) to confirm the in vitro models used in this paper.

## 5. Conclusions

In this study, we highlighted differences in the biological activity of melanomic and melanocytic EVs with regard to stellate cell activation/contractility and endothelial tube formation; however, much remains to be studied about their molecular repertoire before we can identify a specific marker to ease the analysis of EVs released by tumor cells in the blood of UM patients and improve the diagnosis and monitoring of liver metastasis. Since tumor-derived EVs can modify the physiological state of recipient cells and impact the therapeutic resistance, it will be important to determine how UM-derived EVs modulate signaling pathways in hepatic cells, which may ultimately lead to new therapeutic strategies for metastatic UM.

## Figures and Tables

**Table 1:** EV size distribution and concentration determined by NTA. EVs from metastatic (TJU-UM001, Mµ2F), non-metastatic (Mµ2), and healthy cells (NCM, HSteCs) were isolated and analyzed for size (nm) and concentration (particles/mL).

	Cell Types				
EV Size	TJU-UM001	Mµ2F	Mµ2	NCMs	HSteCs
Mean (nm)	212.5 ± 9.2	200.4 ± 5.9	180.0 ± 8.4	96.0 ± 5.6	212.3 ± 2.3
SD (nm)	93.5 ± 8.9	68.1 ± 9.2	70.0 ± 3.6	30.9 ± 2.5	61.0 ± 3.0
* D10 (nm)	84.5 ± 23.0	139.1 ± 13.8	105.5 ± 10.1	56.7 ± 7.6	136.8 ± 7.8
* D50 (nm)	215.9 ± 14.1	193.1 ± 8.7	175.8 ± 12.9	91.8 ± 7.2	217.3 ± 7.4
* D90 (nm)	312.4 ± 12.2	288.7 ± 18.2	262.4 ± 18.2	133.7 ± 6.7	284.2 ± 9.2
Concentration	2.02 × 10^10^	4.88 × 10^9^	1.20 × 10^10^	6.34 × 10^9^	2.34 × 10^10^
(particles/mL)	±3.56 × 10^9^	±1.91 × 10^8^	±9.92 × 10^8^	±4.30 × 10^8^	±8.77 × 10^8^

Particle size distribution D10, D50, and D90 corresponding to the percentages 10%, 50%, 90% of particles under the reported particle size.

## Data Availability

Data are contained within the article or in Appendix.
